# Morphology of Sella Turcica and Bridging Prevalence Correlated with Sex and Craniofacial Skeletal Pattern in Eastern Asia Population: CBCT Study

**DOI:** 10.1155/2021/6646406

**Published:** 2021-03-22

**Authors:** Szu-Ting Chou, Chun-Ming Chen, Ping-Ho Chen, Yuk-Kwan Chen, Shis-Chieh Chen, Yu-Chuan Tseng

**Affiliations:** ^1^School of Dentistry, College of Dental Medicine, Kaohsiung Medical University, Kaohsiung, Taiwan; ^2^Department of Orthodontics, Kaohsiung Medical University Hospital, Kaohsiung, Taiwan; ^3^Department of Oral and Maxillofacial Surgery, Kaohsiung Medical University Hospital, Kaohsiung, Taiwan; ^4^Department of Oral Pathology and Diagnosis, Kaohsiung Medical University Hospital, Kaohsiung, Taiwan

## Abstract

**Purpose:**

Sella turcica bridging (STB) refers to a rare anatomical variation formed by the ossification of the ligament between the anterior and posterior clinoid processes. The presence of the STB was significantly correlated with craniofacial skeleton classification and a higher prevalence rate in skeletal Class III. The current study is aimed at investigating the dimensions of sella turcica and the prevalence of STB in different sexes and on the three craniofacial skeletal patterns using cone beam computed tomography (CBCT).

**Materials and Methods:**

A total of 159 adults (66 males and 93 females), including 3 different craniofacial skeletal patterns (skeletal Classes I, II, and III), were included in the study. The sella turcica dimensions and the prevalence of STB were calculated. An independent *t*-test and generalized linear model were used to compare the differences in the sella turcica dimensions and the skeletal relations. The Spearman rank correlation coefficient was used to analyze the correlations between sella turcica dimensions and skeletal relation.

**Results:**

The sella length (SL) was 11.05 ± 1.80 mm for males and 10.77 ± 1.56 mm for females. The anterior clinoid distance (ACD) which was measured for the anterior width of sella turcica showed 25.83 ± 2.04 mm and 24.04 ± 2.28 mm for males and females, respectively (*p* < 0.0001). The overall percentage of complete bridging and partial bridging was 6.6% and 56.9%, respectively. Complete bridges were more common in males (males: 9.1%, females: 4.8%), and partial bridges were more frequent in females (males: 49.2%, females: 62.4%). Both sexes differed with respect to sella turcica dimensions. Moreover, males had a significantly larger ACD, posterior clinoid distance (PCD), and diameter of sella turcica (DST), on both sides, relative to females.

**Conclusion:**

The prevalence rate of complete STB in the Taiwanese population was 6.6%. Significant differences between sexes were found in sella turcica dimensions. The prevalence rates of STB as well as the sella turcica dimensions did not significantly differ between different craniofacial skeletal patterns (skeletal Classes I, II, and III).

## 1. Introduction

Sella turcica bridge (STB) is a structural variation of the skeletal connection between the anterior clinoid process and the posterior clinoid process; it is a rare phenomenon of ossification of the interclinoid ligament (ICL) [1–4]. Sella point (S point) is an important landmark in cephalometric analysis and is often used for judging craniofacial growth changes and comparing therapeutic effects of skeletal malocclusions achieved by dentofacial orthopedic appliances [5, 6]. The morphology of the cranial base can affect facial aesthetics because it is located near the upper and lower jaws [5].

Most previous studies on the assessment of sella turcica used traditional 2D lateral cephalometric radiographs to determine the existence of STB [7–9], and cephalometric analysis can be affected if an STB is present [10–14]. Although cephalometric analysis is a routine diagnosis tool for orthodontics, its accuracy of STB diagnosis is limited by shortcomings in errors caused by projection, magnification, and overlapping structure images [7, 15]. In addition, the left or right side of the sella turcica cannot be distinguished in lateral cephalometric radiographs, and false positives can result from structure overlapping. Therefore, true bridging (fusion of anterior and posterior clinoid process) is difficult to distinguish from pseudobridging (superposition of ICL) in 2D lateral cephalometric images [16]. Recently, due to the advancement in craniofacial imaging technology, cone beam computed tomography (CBCT) can be used to reconstruct 3D structures [5, 16, 17], thus making the shortcomings of 2D lateral cephalometric images more critical in comparison with 3D CBCT scans. Compared to conventional CT, the technology of CBCT can achieve high-quality images with lower-cost equipment and at a lower radiation dose. Because of these advantages, it is an alternative imaging method for craniofacial structures and the dental field [5, 18, 19].

Incidence rates of 3.8%–13% have been reported in the literature for STB in normal individuals [7, 10, 20, 21]. Patients with craniofacial development anomalies, dental anomalies (including palatal canine impactions) [7, 15], or serious skeletal malocclusion (including skeletal Class III) [20, 22, 23] have increased STB frequency. Becktor et al. [15] observed that among 177 participants with craniofacial development anomalies, 18.6% had STB. Leonardi et al. [7] noted a greater prevalence rate of STB among patients with tooth eruption anomalies. In cases of craniofacial development anomalies or tooth anomalies, 2D studies [7–9] have found apparent increases in STB incidence rates in patients with palatal canine impactions and tooth transpositions. However, using 3D CBCT, Ortiz et al. [16] observed no significant correlation between palatal canine impactions and STB. This result is inconsistent with the other studies that have used 2D X-ray radiographs. Therefore, when CBCT is used to evaluate the spatial structures of sella turcica, the results for STB incidence rate differ from those obtained from 2D X-ray images. Because neural crest cells participate in the formation and development of sella turcica, teeth, and the entire craniofacial area, the various anomalies of this area may be related to each other [7]. The shape variations of the sella turcica area may be caused by aging-related factors (in the normal physiological process) or by the growth and development disorders related to craniofacial or pituitary gland variations. The bridging process of STB is similar to that of ponticulus posticus, which is an abnormal bony bridge in the atlas formed by progressive ossification of the posterior atlanto-occipital ligament and partially or completely encloses the vertebral artery and the first cervical nerve root [24].

Taken together, 3D imaging techniques must be used to reconfirm whether STB is related to different skeletal malocclusions. Although previous 2D studies have observed significant correlations, STB incidence rates were likely to have been overestimated due to the image superposition of X-ray images. In addition, STB incidence rates differ across populations [25, 26]. Reviewing English literature, most studies on sella turcica have been conducted in Western countries with most of them have used 2D X-ray images [11–13, 20] and only a few employed 3D CBCT scans [16]. Furthermore, to our knowledge, there are no CBCT studies on sella turcica for Eastern Asia population. So the purpose of the present study is to use CBCT data to analyze differences in STB incidence rates and sella turcica dimensions across different sexes and skeletal relations in an adult Taiwanese population. The null hypothesis is that (1) there is no significant difference between sexes and (2) there is no significant difference between three skeletal relations.

## 2. Methods and Materials

The current retrospective study was approved by the Institutional Review Board of Kaohsiung Medical University Hospital (KMUHIRB-E(I)-20190346). The imaging data of CBCT were collected in the dental department of our institution from July 2017 to October 2019 using the NewTom VGi evo CBCT machine. The imaging range was set at 24 × 19 mm, and the radiological dose was 110 kV, 4.59 mA. The shooting time was 3.5 s with a voxel of 0.03 mm. In addition, the lateral cephalometric films of the CBCT images were collected, and medical records were reviewed to explicitly document the age, sex, and craniofacial skeletal relations.

Head CBCT files and lateral cephalometric radiographs with clear and diagnosable images aged between 18 and 40 years old were included. Data were excluded for the cases of systemic diseases, severe craniofacial deformity, cleft lip and cleft palate, facial bone trauma, and unclear CBCT scans and lateral cephalometric radiographs.

### 2.1. Sample Classification

Craniofacial skeletal relations were classified into 3 classes according to the A point-nasion-B point (ANB) angle in the cephalometric analysis as follows:

1. Skeletal Class I malocclusion: ANB angle 0–4 degrees
2. Skeletal Class II malocclusion: ANB angle > 4 degrees
3. Skeletal Class III malocclusion: ANB angle < 4 degrees

After preliminary data selection, 159 samples were included (66 men and 93 women). Of the samples, 51 were of Class I malocclusion (19 men and 32 women), 49 were of Class II malocclusion (19 men and 30 women), and 59 were of Class III malocclusion (28 men and 31 women). A compute achieved power as a post hoc was established by effect size as the mean difference of anterior clinoid distance (ACD) between genders, with sample size and alpha level less than 0.05. The power was greater than 0.86, and research results have adequate power.

### 2.2. CBCT Data Processing

**Figure 1:**
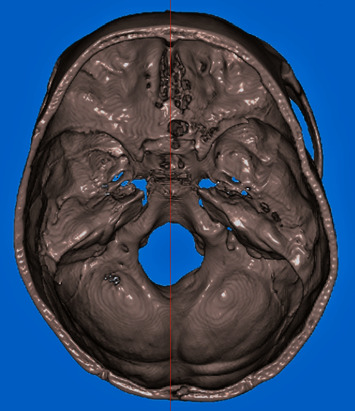
Axial view of sella turcica from top of the head. The red line indicates the midsagittal plane.

**Figure 2:**
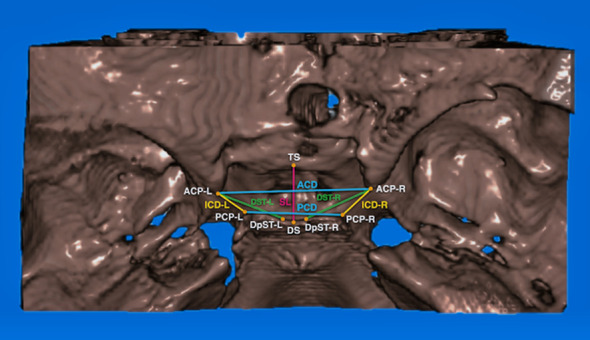
The definitions of the landmarks of sella turcica are listed as follows: (1) tuberculum sella (TS): midpoint on the anterior boundary of sella turcica identified on the midsagittal plane; (2) dorsum sella (DS): midpoint on the posterior boundary of the sella turcica on the midsagittal plane; (3) anterior clinoid process, right (ACP-R): the apex of the anterior clinoid process on the right side; (4) anterior clinoid process, left (ACP-L): the apex of the anterior clinoid process on the left side; (5) posterior clinoid process, right (PCP-R): the apex of the posterior clinoid process on the right side; (6) posterior clinoid process, left (PCP-L): the apex of the posterior clinoid process on the left side; (7) diameter point of sella turcica, right (DpST-R): the furthest point on the inner wall of the pituitary fossa on the right side; (8) diameter point of sella turcica, left (DpST-L): the furthest point on the inner wall of the pituitary fossa on the left side.

**Figure 3:**
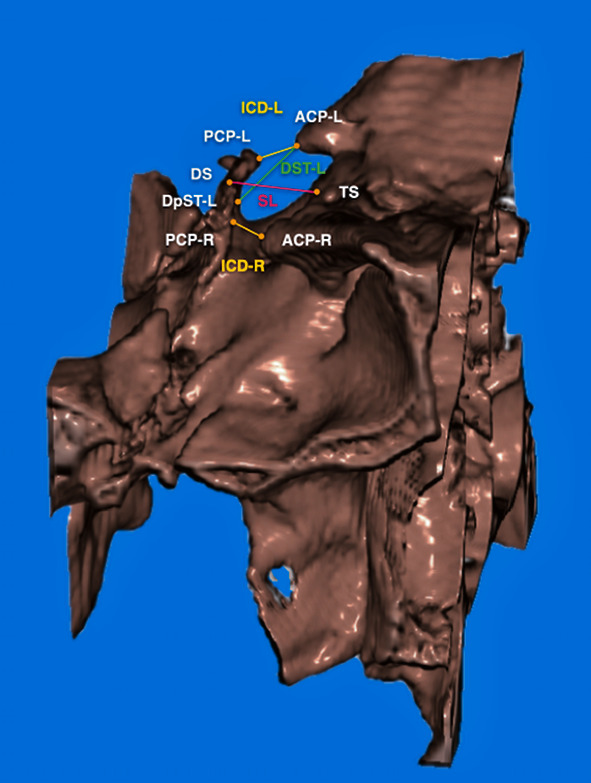
Lateral view of sella turcica from the right side of the head. SL: sella length; ICD-L: interclinoid distance-L; ICD-R: interclinoid distance-R; DST-L: diameter of sella turcica-L.

The DICOM files output by CBCT were reconstructed into 3D structural images (Figures 1–3) using a 3D image editing software program Soteria DcmRecons (version Alpha v0.7.0; Soteria Biotech Ltd., New Taipei City, Taiwan). The Frankfort horizontal planes (porion-right, porion-left, and orbitale-right) were selected and calibrated as axial planes. The planes perpendicular to the axial planes passing through the sella turcica center points were the sagittal planes. In addition, the locations of the left and right orbits and the frontal planes were adjusted. Subsequently, the 3D structural images of sella turcica were captured to obtain linear measurements, such as the sella turcica dimensions (Figures 2 and 3), and sella bridging ratios.

### 2.3. Measurements

**Figure 4:**
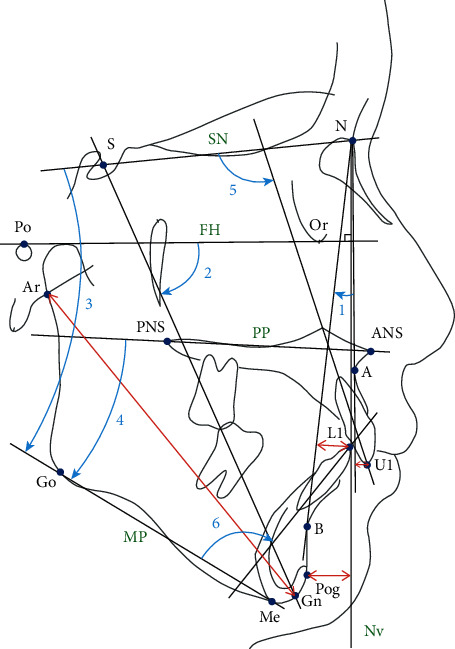
The angular measurements of cephalometric analysis included (1) A point-nasion-B point (ANB) angle; (2) *Y* axis angle, which was the angle formed by the Frankfort horizontal plane (FH plane; Po-Or plane) to the S-Gn plane; (3) SNMP angle (the angle formed by the sella-nasion plane to the mandibular plane or menton-gonion plane); (4) PPMP angle, which was the angle formed by the palatal plane (ANS-PNS plane) to the mandibular plane (menton-gonion plane); (5) U1SN (the angle formed by the long axis of the upper central incisor to sella-nasion plane); (6) L1MP (the angle formed by the long axis of the lower central incisor to the mandibular plane or menton-Gonion plane). The linear measurements of cephalometric analysis are as follows: (1) ArGn (the distance between articulare and gnathion); (2) PogNv (the vertical distance from pogonion to a perpendicular line extended from nasion to the FH plane); (3) U1NA (the distance from the tip of the upper central incisor to nasion-A point line); (4) L1NB (the distance from the tip of lower central incisor to nasion-B point line).

The angular and linear measurements of cephalometric analysis are showed in Figure 4.

The measurements of sella turcica dimensions were recorded as follows (Figures 2 and 3):

1. Sella length (SL) (TS-DS): the distance between tuberculum sella (TS) and dorsum sella (DS)
2. Interclinoid distance-L (ICD-L) (ACP-L to PCP-L): the distance between anterior clinoid process on the left side (ACP-L) and posterior clinoid process on the left side (PCP-L)
3. Interclinoid distance-R (ICD-R) (ACP-R to PCP-R): the distance between anterior clinoid process on the right side (ACP-R) and posterior clinoid process on the right side (PCP-R)
4. Diameter of sella turcica-L (DST-L) (ACP-L to DpST-L): the distance from anterior clinoid process on the left side (ACP-L) to the furthest point on the inner wall of the pituitary fossa on the left side (diameter point of sella turcica, left) (DpST-L)
5. Diameter of sella turcica-R (DST-R) (ACP-R to DpST-R): the distance from anterior clinoid process on the right side (ACP-R) to the furthest point on the inner wall of the pituitary fossa on the right side (diameter point of sella turcica, right) (DpST-R)
6. Anterior clinoid distance (ACD) (ACP-L to ACP-R): the distance between ACP-L and ACP-R. When a complete bridge is presented, the midpoint on the narrowest part of the connection between the tips of the anterior and posterior clinoid processes is selected in order to measure the ACD
7. Posterior clinoid distance (PCD) (PCP-L to PCP-R): the distance between PCP-L and PCP-R
8. Sella turcica bridging ratio-L (STBr-L) (ACP-L to PCP-L/TS-DS) (%): the sella turcica bridging ratio of the left side, which was calculated as ICD-L divided by SL
9. Sella turcica bridging ratio-R (STBr-R) (ACP-R to PCP-R/TS-DS) (%): the sella turcica bridging ratio of the right side, which was calculated as ICD-R divided by SL

The sella turcica bridging ratios were calculated, and the calcification levels of the ICL were quantified using objective quantization methods [16, 27]. The formula is as follows:

(1) $$\begin{array}{c} \text{Sella turcica bridging ratio}=\frac{\text{interclinoid distance}\left(\text{ACP}‐\text{PCP}\right)}{\text{sella length}\left(\text{TS}‐\text{DS}\right)}\times 100\%. \end{array}$$

The STB conditions were divided according to this ratio into complete sella turcica bridge (ratio = 0%), partial sella turcica bridge (0% < ratio < 60%), and no bridge (ratio > 60%).

### 2.4. Statistical Methods

Kappa statistics were used to analyze consistency and reproducibility. Descriptive statistics were used to determine the prevalence rates of complete sella turcica bridge, partial sella turcica bridge, and no bridge. An independent *t*-test and generalized linear model were used to compare the differences in the cephalometric analysis results and sella turcica dimensions. Chi-square was used to calculate the distribution ratios of different ICL connection conditions for the different sexes and craniofacial skeletal relations. Odds ratios were used to discuss the strengths and directions of STB incidence between different sexes. Finally, the Spearman rank correlation coefficient was used to analyze the correlations between each sella turcica dimension and cephalometric analysis value.

## 3. Results

Table 1 presents the differences between male and female samples with respect to age, lateral cephalometric analysis results, and sella turcica dimension. The average age of males was 25.20 ± 4.88 years, and the average age of females was 25.68 ± 5.79 years, with no significant differences in age between the sexes. The average length of sella turcica (sella length (SL)) was 11.05 ± 1.80 mm for males and 10.77 ± 1.56 mm for females. As for the measurement of anterior width of sella turcica, the ACD was 25.83 ± 2.04 mm and 24.04 ± 2.28 mm for males and females, respectively (*p* < 0.0001). The PCD also differed significantly between sexes (*p* < 0.05). The ArGn length was 114.94 ± 10.75 mm for males and 104.79 ± 7.59 mm for females, indicating that the mandible of men was significantly larger than that of women (*p* < 0.0001). The PogNv length was 2.27 ± 12.73 mm for males and significantly smaller at −1.82 ± 10.72 mm for females (*p* = 0.0300). For sella turcica dimensions, ACD, PCD, DST-L, and DST-R were larger for males than females. No significant differences were observed in the other measurements. The three skeletal relations did not significantly differ with respect to sella turcica dimension in males and females. Therefore, the null hypothesis that there is no significant difference between sexes was rejected.

Table 2 demonstrates the differences between the different skeletal relations of both sexes with respect to age, lateral cephalometric analysis results, and sella turcica dimensions. Class I and Class II female samples had significantly older ages than Class III samples (*p* = 0.0175). For linear measurement in ArGn, Class III/male was longer than Class III/female, Class I/male, and Class II/male (*p* < 0.0001). For PogNv, Class III/male and Class III/female were longer than Class I/male; Class I/female was longer than Class II/male and Class II/female (*p* < 0.0001). As for sella turcica dimension, only ACD significantly differed, with Class I/male > Class III/female, Class II/male > Class III/female, and Class III/male > Class III/female. No other category exhibited significant differences between the 6 groups. The null hypothesis that there is no significant difference between three skeletal relations was accepted.

Table 3 indicates the differences between both sexes with respect to sella turcica bridging prevalence. For all complete bridges, 12 were male (57.1%) and 9 were female (42.9%). For males, 9.1%, 49.2%, and 41.7% had a complete bridge, a partial bridge, and no bridge, respectively. For females, the prevalence of complete bridge, partial bridge, and no bridge was 4.8%, 62.4%, and 32.8% respectively. Both sexes significantly differ in comparison to the prevalence of types of sella turcica bridging (*p* = 0.0470). Among all samples, the partial bridge was the most prevalent (56.9%), followed by no bridge (36.5%) and complete bridge (6.6%).

Table 4 presents the differences between the 3 different skeletal relations with respect to sella turcica bridging types. Skeletal Class III has the largest number of complete bridge, more than Class I or Class II.

The percentages of the complete bridge for skeletal Classes III, I, and II were 3.5%, 2.2%, and 0.9%, respectively. The percentages of the partial bridge for skeletal Classes I, II, and III were 19.8%, 17.6%, and 19.5%, respectively. The prevalence rate for types of sella turcica bridging did not differ between the 3 skeletal relations (*p* = 0.2747). The null hypothesis was accepted.

The associations among cephalometric analysis and sella turcica dimensions are showed in Table 5. Within all the linear cephalometric measurements, only ArGn and ACD were positively correlated. In the correlation between different sella turcica dimensions (Table 6), SL was only correlated with ICD-L and ICD-R. Furthermore, ICD-L, ICD-R, DST-L, DST-R, ACD, PCD, STBr-L, and STBr-R all had correlations with each factor.

Each sample was examined by a dentist in accordance with the different sella turcica dimensions. The intraclass correlation coefficient was between 0.776 and 0.985. Based on the 95% confidence interval of the ICC estimate, values less than 0.5, between 0.5 and 0.75, between 0.75 and 0.9, and greater than 0.90 are indicative of poor, moderate, good, and excellent reliability, respectively.

## 4. Discussion

### 4.1. Sella Turcica Bridge Prevalence Rate

The study is the first report of CBCT on sella turcica for the Taiwanese population. This study used CBCT images to determine the existence of STB, and the prevalence of a complete STB was observed to be 6.6%. In human populations, the ossification of ICL is rare; the frequencies of such ossification had been noted to be 4–9% in the literature, with the ossification of a single side more common than that of both sides [28–30]. In this study, the frequency of double-sided STB was 3.77% (6/159) which is less than single side; this finding coincides with previous studies [28–30]. Previous studies have determined that the incidence rate of ICL calcification differs between populations but does not significantly differ between ages [2]. Different incidences of STB have been reported because of differences in the methods and populations of studies. Erturk et al. [31] investigated the Turkish population with adult dry skulls, and through autopsy results, they determined that the incidence rate for an interclinoid bridge was 8.18%. Similarly, Ozdogmus et al. [2] investigating a Turkish population found that the incidence rate for the double-sided complete interclinoid bridge was 6%. In a recent study, Bayrak et al. [32] investigated the incidence rates of physiological intracranial calcification in a Turkish population using CBCT images; they observed ICL calcification in 4.88% of their patients. For the Indian population, the complete calcification ratio of skulls was 4% [4]. In a study of Danish people (i.e., a Caucasian race), Becktor et al. [15] observed an STB incidence rate of 18.6% using lateral cephalometric X-ray radiographs.

### 4.2. Age Difference

The samples in our study were of adults aged 18–40. The results indicated that age was not correlated with the sella turcica dimensions (Table 5). Previous studies have determined that the incidence rate of ICL calcification differs between populations but does not significantly differ between ages [2]. Choi et al. [33] investigated the size and shape of normal sella turcica among 200 Korean orthodontic patients aged 6–42 years. For ages before 25 years, the rate of change of sella turcica dimension, length, depth, and width increased linearly and significantly with age. For ages after 26 years, sella turcica dimension exhibited no significant change. Sella turcica length increased more noticeably than its depth and width. The authors' measured sella turcica dimensions were inconsistent with the results of Axelsson et al. [10] possibly due to differences in the measurement techniques and definitions of anatomical landmarks. A cadaver study of 11–70 years old showed no significant correlation between age and sella turcica length, depth, and sellar opening anteroposterior diameter [34]. However, a CBCT study of Turkish population conducted by Yasa et al. [5] showed that the sella turcica's diameter, depth, length, and the distance between the tips of the anterior clinoid processes vary significantly with age. Gibelli et al. [35] revised 300 computed tomography scans of the head from northern Italian patients and found that sella turcica bridging showed a correlation with age (*p* = 0.007).

### 4.3. Sex Differences

The sexual dimorphism was found in sella turcica dimensions in this study (Table 1). As for sella turcica dimensions in this study, DST-L, DST-R, ACD, and PCD were larger for males than females (Table 1). Among the above measurement items, the most obvious difference between sexes in ACD is presented (*p* < 0.0001) (Tables 1 and 2), suggesting that relative to females, males had a larger-size sella turcica in the anterior and posterior width as well as larger left and right diameters. This finding coincides with that by Akay et al. [36] who used CBCT data and concluded that the distance between anterior clinoid processes differ significantly between sexes. They also reported that interclinoid distance (ICD) in males is significantly larger than that in females in the noncleft group. ICD for males was also larger than that for females in our study; however, it did not achieve statistically significant differences (Table 1).

This result differed from those of previous studies. For example, Alkofide [11] analyzed the shape and size of sella turcica of Saudi Arabian people aged 11–16 years; they observed no significant differences between the sexes with respect to average length, diameter, and depth. Similarly, Moslemzadeh et al. [37] studied Iranian people aged 8–12; they observed that sex had no correlation with the length, depth, and diameter of sella turcica. In addition, Yasa et al. [5] described that there was no significant difference in the measurements including diameter, depth, length, and the interclinoid distance between the tips of the anterior clinoid processes of sella turcica between males and females. Both Alkofide [11] and Yasa et al. [5] confirmed the findings of Lurie et al. [38] in which no significant differences in pituitary gland dimensions between the genders were found by using magnetic resonance imaging (MRI) examination. Anatomical knowledge of the anterior and posterior clinoid processes is essential during neurosurgical operations in order to avoid any damage to structures in relation to the pituitary [5]. The distance between the anterior clinoid processes on both sides has been measured only in a few studies [5].

Other studies have considered sex to affect the dimension of sella turcica. For example, Andredaki et al. [39] studied 184 healthy Greek people aged 6–17, using lateral cephalometric X-ray films, to evaluate the size and shape of sella turcica; they observed that, first, the anterior sella turcica height was taller in females than in males and, second, that females have slightly greater dimension anomalies in the sella turcica. Axelsson et al. [10] noted longer sella turcica length in males than in females, but almost identical depth and greatest diameter, during the development process in their longitudinal period of observation (6–21 years old). For the influence of sex on the size of sella turcica, data have been differed between studies, implicating this influence has been undetermined in the literature.

In our study, a complete bridge was more common in males (9.1%) than females (4.8%) (Table 3), and a partial bridge was significantly more common in females (62.4%) than in males (49.2%) (*p* = 0.0470). This result differs from those in previous studies. For example, Cederberg et al. [1] investigated a US population and noted 8% and 39% frequency in calcification and incomplete calcification, respectively; these frequencies were weakly correlated with age and not correlated with sex. Leonardi et al. [7] had similar results, where “ICL calcification” (STB) was neither related to sex nor age.

In our study, the cephalometric differences between men and women occur only in skeletal characteristics of the mandible, including ArGn, PogNv, and SNMP (Table 1). These results show that men have longer mandibular length, more protrusive mandibular position, and smaller mandibular plane angle.

### 4.4. Craniofacial Skeletal Relationships

The results of this CBCT study indicated that the three skeletal relations did not significantly differ with respect to sella turcica dimensions except the width of ACD (Table 2). Also, the prevalence rate for types of sella turcica bridging did not differ between the three skeletal relations (*p* = 0.2747) (Table 4). This result differs from those in the literature using lateral cephalometric X-ray films [11–13, 15, 20, 22, 23, 37]. For example, Becktor et al. [15] noted that compared with normal individuals, patients with severe craniofacial deviations had greater STB incidence rates. Furthermore, Jones et al. [20] noted that compared with an orthodontics-only group, a combined surgical-orthodontic group had significantly larger average area and perimeter in their sella turcica and a significantly smaller average interclinoid distance (ICD). These results indicated a higher probability of STB and abnormal sella turcica sizes in the combined surgical-orthodontic group. Sobuti et al. [13] investigated 105 Iranian patients aged 14–26 using lateral cephalometric X-ray images, analyzing the sella turcica dimensions and STB incidence rates for cases of Class I, II, and III skeletal malocclusions. They noted that STB was significantly correlated with craniofacial skeleton classification, with the incidence rate of STB being higher in skeletal Class III. Sathyanarayana et al. [12] also noted higher STB incidence rates and larger sizes for Class III relation relative to their Class I and Class II counterparts. Marsan and Öztas [22] investigated Turkish adult female with Class I or Class III relation; they noted a higher STB prevalence rate in the Class III group but no significant differences between the classes with respect to sella turcica dimension. Meyer-Marcotty et al. [23] evaluated patients older than 17 years of age with Class I or Class III relation; they noted a higher STB prevalence rate in skeletal Class III. In our study, a higher percentage of complete bridge was noted in skeletal Class III; however, there was no significant difference between skeletal Classes I, II, and III (Table 4).

Differences between the sexes were analyzed with respect to the three craniofacial skeletal relations (Table 2). As for sella turcica dimension, only ACD significantly differed between the 6 groups (comprising 3 skeletal classes for both genders), where Class I/male was longer than Class III/female, Class II/male was longer than Class III/female, and Class III/male was longer than Class III/female (Table 2). These differences in the ACD may be due to differences in sex rather than differences in skeletal relations (Tables 1 and 2). This result differs from that of Alkofide et al. [11], in which Class II and Class III significantly differed with respect to the sella turcica dimension. Patients with a Class III skeletal relation have larger sella turcica diameters, and Class II patients have apparently smaller sella turcica diameters [11]. The sella length (SL) measured in our study did not show significant difference between three skeletal malocclusions (Table 2). This result did not coincide with that of Moslemzadeh et al. [37] who investigated 108 Iranians' lateral cephalometric X-ray films (36 Class I, 36 Class II, and 36 Class III) and concluded that the sella turcica length significantly differed between the Class I and Class III groups, in which the Class III group had a larger sella turcica length.

### 4.5. Difference between 2D Lateral Cephalograms and 3D CBCT

CBCT is an imaging technique widely applied in dental diagnosis and treatment. Intracranial physiological calcifications are common accidental discoveries in CBCT scans. Compared with CT, CBCT affords more sensitive, specific, and superior imaging of the anatomical structure, particularly when identifying intracranial physiological calcification. CBCT images can effectively study the anatomical structure of the sella turcica; in addition, the information of the linear dimensions and shape of sella turcica can be used as reference standards for clinical investigations [5]. The relation between skeletal malocclusion and other structural characteristics such as the mandibular condyle can also be studied more easily in CBCT scans than in 2D lateral cephalograms. In a recent CBCT study by Lo Giudice et al. [40], the significant differences in condylar cortical bone thickness were found between different vertical facial dimensions.

In the present study, the STB prevalence rates of different skeletal patterns did not significantly differ, different from the results of previous studies. Because this study used 3D images that were reconstructed from CBCT data, sella turcica dimensions were measured in detail, complete STB were correctly determined, and the distributions of STB on the left and right sides were documented. Therefore, this study avoided the limitations of previous studies, which used 2D X-ray films [11–13, 15, 20, 22, 23, 37]. These disadvantages include limitations in radiation angles when diagnosing STB, errors caused by magnifications as well as the overlapping of left and right structure images, and the mistaken identification of pseudobridging (ICL superposition) as true bridging (fusion of anterior clinoid process and posterior clinoid process) [16]. In addition, because STB can appear in different forms, in previous 2D studies [7, 9], the conditions of the bridge of the middle clinoid processes could have been mistaken to be STB (connection of anterior clinoid process and posterior clinoid process), resulting in the overestimation of STB incidence rates [16]. Because of these differences in method, the findings of the aforementioned studies cannot be accurately compared. Furthermore, differences between the populations of study can also result in differences in the study results. The differences between studies may be due to the fact that different landmarks represent the same dimension, superimposition of related anatomic structures, different degree of magnification, and the difference in the composition of study samples [5].

Up to date, only two studies [36, 41] have studied the sella turcica dimensions and shape via CBCT specially focused at different skeletal relations. Akay et al. [36] reported that interclinoid distance and dimensions of sella turcica did not differ significantly in different skeletal relation in Turkey subjects. Silveira et al. [41] studied differences only between Class II and III relations of Brazil patients and indicated that there is no significant difference in size of anterior cranial base between Classes II and III, but large size of anterior cranial base in male subjects was founded. Neither of these two studies have mentioned about the analysis of STB.

The 3D structure of STB can be observed using CBCT. Because the phenomenon of superposition is absent, the diagnosis of complete bridging is easier. However, the distinguishing of partial bridge from no bridge sella turcica is considered difficult. In their anatomical studies of human dry skulls, Archana et al. [4] and Kolagi et al. [26] faced similar difficulties when using direct observation to classify STB, particularly when distinguishing a partial bridge from the absence of a bridge, potentially causing measurement errors. In addition, the shapes and forms of the clinoid processes vary widely [10] and the positions of the terminal points of clinoid processes as well as starting points of bridges are usually unclear. Therefore, to reduce the possibility of misjudgment due to either differences in classification standards or difficulties in identification, an objective quantification method must be adopted to determine the type of STB.

## 5. Limitations

In the current study, only limited samples were collected from July 2017 to October 2019 for analysis and there may still be some inconsistencies with the general population. Since subjects included in this study were not from the population but from the university dental clinic, there may be some inherent bias [6]. Future studies should increase the sample size to validate the relationship between STB and different sex and craniofacial skeletal patterns. In addition, only those aged between 18 and 40 were included in the study, so it was impossible to assess the condition of children and adolescent patients in the current study. Also, during the evaluation of CBCT data for studies making small measurements, the limitations such as spatial resolution and the evaluation of bone density should be considered [42].

In conclusion, the study is the first report of CBCT on sella turcica for Eastern Asia population. The current study used CBCT images of a Taiwanese population to determine the existence of STB, and the prevalence rate of complete STB was 6.6%. The prevalence rates of STB as well as the sella turcica dimensions did not significantly differ between different craniofacial skeletal patterns (skeletal Classes I, II, and III). Both sexes differed with respect to sella turcica dimensions. In particular, males had a significantly larger ACD, PCD, DST-L, and DST-R, relative to female individuals. Conversely, no significance was discovered in other measurements. Complete bridges were more common in males (males: 9.1%, females: 4.8%), and partial bridges were more common in female individuals (males: 49.2%, females: 62.4%). For ACD, males were larger than that for females in skeletal Classes I, II, and III. The mandibular length (ArGn) and ACD were positively correlated.

## Figures and Tables

**Table 1:** Cephalometric analysis and sella turcica dimensions of all samples by sex.

Cephalometric analysis and sella turcica dimensions	Male (**n** = 66)				Female (**n** = 93)				**p** value
	Mean	SD	Min	Max	Mean	SD	Min	Max	
Age (year)	25.20	4.88	18.00	39.00	25.68	5.79	18.00	39.00	0.5850
ANB (°)	0.59	5.68	-14.80	11.00	1.83	5.06	-8.70	11.90	0.1510
ArGn (mm)	114.94	10.75	75.80	133.60	104.79	7.59	84.00	119.30	<0.0001^∗^
PogNv (mm)	2.27	12.73	-22.50	27.70	-1.82	10.72	-25.30	18.20	0.0300^∗^
**Y** axis (°)	68.41	5.65	52.80	81.30	70.32	7.87	23.00	84.70	0.0930
SNMP (°)	33.16	7.06	10.40	58.10	35.83	7.72	16.70	56.70	0.0280^∗^
PPMP (°)	23.06	6.80	6.30	41.80	25.25	7.42	5.50	46.00	0.0590
U1SN (°)	109.65	9.68	83.70	132.20	107.97	9.57	82.70	128.30	0.2770
U1NA (mm)	6.68	3.15	-1.80	13.80	6.83	2.72	-2.20	11.70	0.7400
L1MP (°)	90.55	12.00	56.30	112.70	92.44	10.85	64.80	118.70	0.3030
L1NB (mm)	7.52	3.76	-3.80	16.20	7.70	3.75	0.10	17.30	0.7780
SL	11.05	1.80	7.07	15.39	10.77	1.56	6.60	14.20	0.3050
ICD-L	6.03	2.86	0.00	13.40	5.68	2.30	0.00	11.36	0.3960
ICD-R	5.36	2.51	0.00	9.70	5.04	2.34	0.00	9.63	0.4040
DST-L	12.14	1.69	7.57	16.98	11.58	1.61	6.85	14.67	0.0340^∗^
DST-R	12.01	1.44	8.03	15.24	11.49	1.54	8.45	15.40	0.0310^∗^
ACD	25.83	2.04	20.63	29.56	24.04	2.28	19.70	30.08	<0.0001^∗^
PCD	17.76	3.11	11.43	24.97	16.78	2.83	9.48	23.54	0.0420^∗^
STBr-L	55.23%	25.67%	0.00%	99.86%	52.52%	20.00%	0.00%	91.93%	0.4560
STBr-R	49.56%	24.60%	0.00%	113.42%	46.69%	20.87%	0.00%	84.97%	0.4280

^∗^ *p* < 0.05; SL: sella length; ICD-L: interclinoid distance-L; ICD-R: interclinoid distance-R; DST-L: diameter of sella turcica-L; DST-R: diameter of sella turcica-R; ACD: anterior clinoid distance; PCD: posterior clinoid distance; STBr-L: sella turcica bridging ratio-L; STBr-R: sella turcica bridging ratio-R.

**Table 2:** Cephalometric analysis and sella turcica dimensions between skeletal groups and sex.

Cephalometric analysis and sella turcica dimensions	Class I				Class II				Class III				*F*	*p* value	Significant
	Male (*n* = 19)		Female (*n* = 32)		Male (*n* = 19)		Female (*n* = 30)		Male (*n* = 28)		Female (*n* = 31)				
	Mean	SD	Mean	SD	Mean	SD	Mean	SD	Mean	SD	Mean	SD			
Age (year)	24.97	4.88	26.99	6.38	26.83	5.46	26.95	5.05	24.26	4.35	23.10	5.06	2.841	0.0175^∗^	IF, IIF > IIIF
ANB (°)	2.00	1.12	1.91	1.21	7.25	1.91	7.68	2.08	-4.88	3.27	-3.90	2.18	169.288	<0.0001^∗^	IIF, IIM > IM, IF > IIIF, IIIM
ArGn (mm)	110.87	10.48	104.73	5.84	106.74	7.16	98.06	5.61	123.26	6.31	111.36	4.60	47.444	<0.0001^∗^	IIIM > IIIF, IM, IIM IM, IIM > IIM, IF > IIF
PogNv (mm)	-0.49	7.29	-1.04	6.56	-11.60	6.01	-13.48	6.10	13.56	7.59	8.67	4.68	76.233	<0.0001^∗^	IIIM, IIIF > IM, IF > IIM, IIF
*Y* axis (°)	69.27	2.96	68.42	9.10	74.00	4.12	77.34	3.98	64.03	4.16	65.49	3.41	25.576	<0.0001^∗^	IIF, IIM > IM, IF, IIIF IM, IF > IIIM
SNMP (°)	36.65	7.05	35.08	6.85	34.26	6.96	41.17	7.31	30.06	5.92	31.43	5.81	10.327	<0.0001^∗^	IIF > IF, IIM, IIIF, IIIM IM > IIIIM
PPMP (°)	26.33	7.07	23.95	6.41	23.01	6.97	30.20	7.53	20.88	5.76	21.81	5.74	7.815	<0.0001^∗^	IIF > IF, IIM, IIIF, IIIM
U1SN (°)	110.39	6.32	108.03	8.42	101.17	9.54	101.04	8.18	114.91	7.65	114.60	7.04	16.203	<0.0001^∗^	IIIM, IIIF > IF > IIM, IIF IM > IIM, IIF
U1NA (mm)	6.15	1.73	6.81	2.76	4.11	3.04	5.48	2.89	8.77	2.53	8.17	1.74	11.570	<0.0001^∗^	IIIM > IF > IM, IF, IIF > IIM IIIF > IIF, IIM
L1MP (°)	91.98	5.93	93.58	10.83	102.80	6.44	100.97	5.83	81.28	9.97	83.01	6.40	32.142	<0.0001^∗^	IIM, IIF > IF, IM > IIIF, IIIM
L1NB (mm)	8.06	2.68	7.43	2.59	10.24	3.27	10.95	3.32	5.31	3.41	4.82	2.52	19.472	<0.0001^∗^	IIF > IF > IM, IF > IIIF, IIIM IIM > IF > IIIF, IIIM
SL	11.22	1.73	10.45	1.56	11.42	1.85	10.81	1.64	10.68	1.82	11.07	1.45	1.164	0.3292	
ICD-L	5.78	3.04	5.34	2.40	7.08	1.78	5.78	1.98	5.48	3.19	5.93	2.52	1.280	0.2754	
ICD-R	5.28	2.24	5.07	2.09	5.61	2.67	4.85	2.46	5.24	2.65	5.19	2.52	0.257	0.9357	
DST-L	12.33	1.62	11.51	1.80	12.27	2.09	11.80	1.54	11.94	1.45	11.42	1.49	1.238	0.2942	
DST-R	12.02	1.33	11.57	1.71	12.18	1.74	11.61	1.72	11.90	1.31	11.29	1.16	1.172	0.3253	
ACD	25.87	2.34	24.26	2.08	26.09	2.25	24.45	2.55	25.62	1.70	23.41	2.13	6.110	<0.0001^∗^	IM > IIIF IIM > IIIF IIIM > IIIF
PCD	18.62	3.00	17.33	2.93	16.99	2.29	16.59	2.60	17.69	3.59	16.41	2.96	1.771	0.1220	
STBr-L	0.52	0.26	0.51	0.21	0.63	0.16	0.54	0.17	0.52	0.30	0.53	0.22	0.763	0.5776	
STBr-R	0.48	0.20	0.48	0.19	0.50	0.24	0.45	0.22	0.51	0.28	0.47	0.23	0.230	0.9490	

^∗^ *p* < 0.05; SL: sella length; ICD-L: interclinoid distance-L; ICD-R: interclinoid distance-R; DST-L: diameter of sella turcica-L; DST-R: diameter of sella turcica-R; ACD: anterior clinoid distance; PCD: posterior clinoid distance; STBr-L: sella turcica bridging ratio-L; STBr-R: sella turcica bridging ratio-R; IM: Class I male; IIM: Class II male; IIIM: Class III male; IF: Class I female; IIF: Class II female; IIIF: Class III female.

**Table 3:** Frequency of sella turcica bridging by sex.

Bridging		Sex		Total	*χ* ^2^	df	**p**
		Male	Female				
Complete bridge (**ratio** = 0)	Count	12	9	21	6.116	2	0.0470^∗^
	% within bridge	57.1%	42.9%	100.0%			
	% within sex	9.1%	4.8%	6.6%			
	% of total	3.8%	2.8%	6.6%			

Partial bridge (**ratio** > 0 and <60%)	Count	65	116	181			
	% within bridge	35.9%	64.1%	100.0%			
	% within sex	49.2%	62.4%	56.9%			
	% of total	20.4%	36.5%	56.9%			

No bridge (**ratio** ≥ 60%)	Count	55	61	116			
	% within bridge	47.4%	52.6%	100.0%			
	% within sex	41.7%	32.8%	36.5%			
	% of total	17.3%	19.2%	36.5%			

Total	Count	132	186	318			
	% within bridge	41.5%	58.5%	100.0%			
	% within sex	100.0%	100.0%	100.0%			
	% of total	41.5%	58.5%	100.0%			

Sella turcica bridging based on 60% cut-off (Camp, 1923). *χ*^2^: chi-square value; df: degree of freedom; *p*: *p* value. Statistically significant at *p* < 0.05. ^∗^*p* < 0.05.

**Table 4:** Frequency of sella turcica bridging by skeletal groups.

Bridging		Class			Total	*χ* ^2^	df	**p**
		Class I	Class II	Class III				
Complete bridge (**ratio** = 0)	Count	7	3	11	21	5.125	4	0.2747
	% within bridge	33.3%	14.3%	52.4%	100.0%			
	% within class	6.9%	3.1%	9.3%	6.6%			
	% of total	2.2%	0.9%	3.5%	6.6%			

Partial bridge (**ratio** > 0 and <60%)	Count	63	56	62	181			
	% within bridge	34.8%	30.9%	34.3%	100.0%			
	% within class	61.8%	57.1%	52.5%	56.9%			
	% of total	19.8%	17.6%	19.5%	56.9%			

No bridge (**ratio** ≥ 60%)	Count	32	39	45	116			
	% within bridge	27.6%	33.6%	38.8%	100.0%			
	% within class	31.4%	39.8%	38.1%	36.5%			
	% of total	10.1%	12.3%	14.2%	36.5%			

Total	Count	102	98	118	318			
	% within bridge	32.1%	30.8%	37.1%	100.0%			
	% within class	100.0%	100.0%	100.0%	100.0%			
	% of total	32.1%	30.8%	37.1%	100.0%			

Sella turcica bridging based on 60% cut-off (Camp, 1923). *χ*^2^: chi-square value; df: degree of freedom; *p*: *p* value. Statistically significant at *p* < 0.05.

**Table 5:** Associations among cephalometric analysis and sella turcica dimensions and the Pearson correlation coefficient.

	SL	ICD-L	ICD-R	DST-L	DST-R	ACD	PCD	STBr-L	STBr-R
Age	-0.048	-0.093	-0.091	-0.009	-0.039	-0.130	0.047	-0.071	-0.072
ANB (°)	-0.007	0.074	-0.038	0.057	0.054	0.044	-0.038	0.081	-0.051
ArGn	0.063	-0.009	0.090	0.020	0.043	0.214^∗∗^	0.141	-0.030	0.099
PogNv	0.021	-0.073	0.060	-0.089	-0.075	-0.055	0.033	-0.079	0.078
*Y* axis (°)	0.143	0.169^∗^	-0.051	0.062	-0.003	-0.027	-0.085	0.121	-0.114
SNMP (°)	0.092	0.104	-0.116	-0.003	-0.062	-0.065	-0.019	0.064	-0.167^∗^
PPMP (°)	0.073	-0.011	-0.202^∗^	-0.064	-0.116	-0.094	0.079	-0.043	-0.242^∗∗^
U1SN (°)	-0.060	-0.059	-0.005	-0.044	-0.084	0.048	0.037	-0.025	0.039
U1NA	-0.031	0.006	-0.092	-0.011	-0.096	0.041	0.088	0.032	-0.059
L1MP (°)	-0.052	0.067	0.029	0.151	0.114	0.172^∗^	0.003	0.099	0.040
L1NB	-0.034	0.093	-0.026	0.186^∗^	0.071	0.206^∗∗^	0.068	0.108	-0.023

^∗∗^Correlations significant at the 0.01 level (2-tailed). ^∗^Correlations significant at the 0.05 level (2-tailed). SL: sella length; ICD-L: interclinoid distance-L; ICD-R: interclinoid distance-R; DST-L: diameter of sella turcica-L; DST-R: diameter of sella turcica-R; ACD: anterior clinoid distance; PCD: posterior clinoid distance; STBr-L: sella turcica bridging ratio-L; STBr-R: sella turcica bridging ratio-R.

**Table 6:** Associations among different sella turcica dimensions and the Pearson correlation coefficient.

	SL	ICD-L	ICD-R	DST-L	DST-R	ACD	PCD	STBr-L	STBr-R
SL	1	0.301^∗∗^	0.186^∗^	0.141	0.064	0.001	-0.061	-0.047	-0.112
ICD-L		1	0.654^∗∗^	0.587^∗∗^	0.407^∗∗^	0.310^∗∗^	-0.599^∗∗^	0.925^∗∗^	0.556^∗∗^
ICD-R			1	0.470^∗∗^	0.585^∗∗^	0.375^∗∗^	-0.592^∗∗^	0.612^∗∗^	0.942^∗∗^
DST-L				1	0.609^∗∗^	0.533^∗∗^	-0.169^∗^	0.557^∗∗^	0.430^∗∗^
DST-R					1	0.510^∗∗^	-0.163^∗^	0.411^∗∗^	0.582^∗∗^
ACD						1	0.183^∗^	0.338^∗∗^	0.391^∗∗^
PCD							1	-0.608^∗∗^	-0.577^∗∗^
STBr-L								1	0.636^∗∗^
STBr-R									1

^∗∗^Correlations significant at the 0.01 level (2-tailed). ^∗^Correlations significant at the 0.05 level (2-tailed). SL: sella length; ICD-L: interclinoid distance-L; ICD-R: interclinoid distance-R; DST-L: diameter of sella turcica-L; DST-R: diameter of sella turcica-R; ACD: anterior clinoid distance; PCD: posterior clinoid distance; STBr-L: sella turcica bridging ratio-L; STBr-R: sella turcica bridging ratio-R.

## Data Availability

The datasets used and/or analyzed during the current study are available from the corresponding author on reasonable request.

## Abbreviations

STB: Sella turcica bridge
ICL: Interclinoid ligament
CBCT: Cone beam computed tomography
SL: Sella length
ICD-L: Interclinoid distance-L
ICD-R: Interclinoid distance-R
DST-L: Diameter of sella turcica-L
DST-R: Diameter of sella turcica-R
ACD: Anterior clinoid distance
PCD: Posterior clinoid distance
STBr-L: Sella turcica bridging ratio-L
STBr-R: Sella turcica bridging ratio-R.
